# Autophagy Inhibition Enhances the Antitumor Efficacy of MET Targeting in MET-High Pancreatic Cancer

**DOI:** 10.32604/or.2026.084396

**Published:** 2026-09-14

**Authors:** Zhiyi Min, Chunbin Wang, Tongjin Yin, Wanyan Jiao, Dandan Zhou, Xuyao Zhang, Junli Cui, Zhe Ding

**Affiliations:** 1 Department of Pharmacy, Yancheng Third People’s Hospital (The Affiliated Hospital of Jiangsu Medical College and the Affiliated Hospital 6 of Nantong University), Yancheng, China; 2 Department of Biological Medicines and Shanghai Engineering Research Center of Immunotherapeutics, Fudan University School of Pharmacy, Shanghai, China; 3 Department of Oncology, Yancheng Third People’s Hospital (The Affiliated Hospital of Jiangsu Medical College and the Affiliated Hospital 6 of Nantong University), Yancheng, China; 4 Department of Pediatrics, Yancheng Third People’s Hospital (The Affiliated Hospital of Jiangsu Medical College and the Affiliated Hospital 6 of Nantong University), Yancheng, China; 5 Department of Pharmacy, The Affiliated Dongtai Hospital of Nantong University, Yancheng, China

**Keywords:** Savolitinib, pancreatic cancer, MET, autophagy, combination therapy

## Abstract

**Objectives:** MET inhibitors have demonstrated clinical efficacy in several MET-driven malignancies; however, their therapeutic potential in pancreatic cancer remains insufficiently characterized. This study aimed to evaluate the antitumor activity of the selective MET inhibitor savolitinib in MET-high pancreatic cancer and to investigate the role of autophagy in the cellular response to MET inhibition. **Methods:** MET-high pancreatic cancer cell lines (AsPC-1 and BxPC-3) were treated with savolitinib. Cell viability, apoptosis, transcriptomic profiling, and signaling pathway analyses were performed to characterize its antitumor effects and underlying mechanisms. Autophagy induction was assessed using monodansylcadaverine (MDC) staining, transmission electron microscopy, lysosomal co-localization analysis, and western blotting of autophagy-related markers. The therapeutic efficacy of combining savolitinib with the autophagy inhibitor chloroquine was further evaluated *in vitro* and in BxPC-3 xenograft models. **Results:** Savolitinib significantly inhibited MET phosphorylation and reduced the viability of MET-high pancreatic cancer cells while inducing apoptosis, as evidenced by increased levels of cleaved PARP, cleaved caspase-3, and an elevated Bcl-2-associated X protein (Bax)/B-cell lymphoma 2 (Bcl-2) ratio. Transcriptomic analysis revealed significant enrichment of autophagy-related pathways among savolitinib-responsive genes. Savolitinib induced autophagy-associated vesicular changes and autophagic flux, accompanied by suppression of AKT/mTOR signaling. Pharmacological inhibition of autophagy with chloroquine markedly enhanced savolitinib-induced apoptosis *in vitro* and significantly improved tumor growth inhibition *in vivo*, achieving a tumor growth inhibition rate of 84.96% in the combination-treatment group without obvious systemic toxicity under the tested conditions. **Conclusion:** Savolitinib-induced autophagy functions as an adaptive cytoprotective response in MET-high pancreatic cancer. Combined inhibition of MET and autophagy enhances antitumor activity and warrants further evaluation in MET-high pancreatic cancer models.

## 1 Introduction

Pancreatic ductal adenocarcinoma (PDAC) is among the most aggressive solid malignancies and remains a major cause of cancer-related mortality worldwide [1]. Owing to its asymptomatic progression, most patients are diagnosed at advanced or metastatic stages, limiting the opportunity for curative surgical intervention. Although chemotherapy-based regimens have modestly improved clinical outcomes, the overall prognosis remains poor, with a 5-year survival rate of approximately 10–13% [1,2]. Given the limited efficacy of current therapeutic options, the identification of actionable molecular targets represents an important strategy for improving patient outcomes [3].

Among the signaling pathways involved in PDAC progression, the hepatocyte growth factor (HGF)/MET axis has emerged as a biologically and therapeutically relevant pathway. MET is a receptor tyrosine kinase encoded by the MET proto-oncogene and is primarily activated by its ligand HGF [4]. Upon HGF binding, MET undergoes receptor dimerization and autophosphorylation, thereby recruiting intracellular adaptor proteins and activating multiple downstream signaling cascades, including PI3K/AKT/mTOR, RAS/MAPK, and STAT3 [4,5]. Through these pathways, MET signaling regulates key malignant phenotypes, such as tumor cell proliferation, survival, migration, invasion, angiogenesis, and metastatic dissemination. Aberrant MET activation may occur through gene amplification, activating mutations, exon 14 skipping, enhanced ligand-dependent stimulation, or protein overexpression, resulting in sustained oncogenic signaling and therapeutic resistance [4]. In PDAC, elevated MET expression has been reported and is associated with aggressive tumor behavior, resistance to chemotherapy, metastatic potential, and unfavorable clinical outcomes [6,7]. These findings support MET as a rational therapeutic target in MET-high PDAC.

The therapeutic relevance of MET dysregulation has led to the development of several MET-targeted strategies, including monoclonal antibodies and small-molecule tyrosine kinase inhibitors [4,8]. However, the biological effects of MET inhibition may vary according to tumor type, MET alteration status, and inhibitor selectivity. Savolitinib is a potent and highly selective MET tyrosine kinase inhibitor designed to suppress MET-dependent signaling while minimizing off-target kinase activity. Compared with multi-target kinase inhibitors such as crizotinib, which also inhibits ALK and ROS1, savolitinib provides a more selective pharmacological approach for investigating MET-dependent tumor cell survival [9,10]. Savolitinib has demonstrated antitumor activity in MET-driven malignancies, particularly non-small cell lung cancer harboring MET exon 14 skipping alteration [11] and MET-amplified gastric cancer [12,13]. Nevertheless, its therapeutic potential in MET-high PDAC has not been fully characterized, and the adaptive cellular responses triggered by selective MET inhibition remain insufficiently understood.

Autophagy is a conserved lysosome-dependent degradation process that maintains cellular homeostasis by recycling damaged organelles and macromolecules. In cancer, autophagy has a context-dependent role: it can suppress tumor initiation by limiting cellular damage, but in established tumors it may also function as a survival mechanism that enables cancer cells to adapt to nutrient deprivation, hypoxia, metabolic stress, and anticancer therapy [14]. Various targeted therapies have been reported to induce protective autophagy, thereby reducing drug-induced cytotoxicity and contributing to therapeutic resistance [14,15]. Notably, the PI3K/AKT/mTOR signaling axis is both a major downstream effector of MET signaling and a central negative regulator of autophagy initiation. Sustained AKT/mTOR activation suppresses autophagy, whereas inhibition of this pathway can relieve mTOR-mediated repression and promote autophagic activity [16]. We therefore hypothesized that selective MET inhibition by savolitinib may suppress AKT/mTOR signaling and induce adaptive autophagy in MET-high PDAC cells. We further asked whether this autophagic response functions as a cytoprotective mechanism and whether autophagy inhibition could enhance the antitumor efficacy of MET-targeted therapy.

In this study, we aimed to evaluate the antitumor activity of savolitinib in MET-high PDAC models and to elucidate the role of adaptive autophagy in the cellular response to selective MET inhibition. We first evaluated the effects of savolitinib on MET phosphorylation, downstream signaling, cell viability, and apoptosis in PDAC cell lines with different levels of MET expression. We then examined whether savolitinib induced autophagy and characterized the involvement of the AKT/mTOR pathway in this process. Finally, we determined the functional role of savolitinib-induced autophagy and assessed whether pharmacological autophagy inhibition with chloroquine could enhance the antitumor efficacy of savolitinib both *in vitro* and *in vivo*.

## 2 Materials and Methods

### 2.1 Reagents and Antibodies

The following reagents were used in this study: savolitinib (S413809; Shanghai Aladdin Biochemical Technology Co., Ltd., Shanghai, China), chloroquine diphosphate (S4157; Selleck Chemicals, Houston, TX, USA), rapamycin (S115842; Shanghai Aladdin Biochemical Technology Co., Ltd., Shanghai, China), Annexin V-fluorescein isothiocyanate/propidium iodide (Annexin V-FITC/PI) Apoptosis Detection Kit (MA0220; Dalian Meilun Biotechnology Co., Ltd., Dalian, China), Autophagy Staining Assay Kit with monodansylcadaverine (MDC) (C3018S; Beyotime Biotechnology, Shanghai, China), Hoechst 33342 staining solution (MA0126; Dalian Meilun Biotechnology Co., Ltd.), and LysoTracker Red DND-99 (L7528; Thermo Fisher Scientific, Waltham, MA, USA). Histological processing, hematoxylin and eosin (H&E) staining, and 3,3′-diaminobenzidine (DAB)-based immunohistochemistry were performed by Servicebio Technology Co., Ltd. (Wuhan, China) according to standardized procedures.

The following primary antibodies were used for western blotting and immunohistochemistry. For western blotting, primary antibodies were generally diluted 1:1000, unless otherwise indicated, and horseradish peroxidase (HRP)-conjugated secondary antibodies were diluted 1:5000: anti-MET antibody (25869-1-AP; Proteintech Group, Inc., Wuhan, China; 1:500), anti-phospho-MET antibody (30737-1-AP; Proteintech Group, Inc.), anti-cleaved PARP antibody (60555-1-Ig; Proteintech Group, Inc.), anti-cleaved caspase-3 antibody (9661; Cell Signaling Technology, Danvers, MA, USA), anti-Bax antibody (50599-2-Ig; Proteintech Group, Inc.), anti-Bcl-2 antibody (12789-1-AP; Proteintech Group, Inc.), anti-p62/SQSTM1 antibody (18420-1-AP; Proteintech Group, Inc.), anti-LC3 antibody (4108; Cell Signaling Technology), anti-mTOR antibody (20657-1-AP; Proteintech Group, Inc.), anti-phospho-mTOR antibody (67778-1-Ig; Proteintech Group, Inc.), anti-p70S6K antibody (14485-1-AP; Proteintech Group, Inc.), anti-phospho-p70S6K antibody (9208; Cell Signaling Technology), anti-AKT antibody (10176-2-AP; Proteintech Group, Inc.), anti-phospho-AKT antibody (4060; Cell Signaling Technology), anti-4E-BP1 antibody (60246-1-Ig; Proteintech Group, Inc.), anti-phospho-4E-BP1 antibody (2855; Cell Signaling Technology), anti-glyceraldehyde-3-phosphate dehydrogenase (GAPDH) antibody (60004-1-Ig; Proteintech Group, Inc.), anti-Ki-67 antibody (GB121499-100; Servicebio Technology Co., Ltd., Wuhan, China; 1:500) and anti-proliferating cell nuclear antigen (PCNA) antibody (GB11010; Servicebio Technology Co., Ltd.; 1:500).

### 2.2 Cell Viability Assays

Human pancreatic ductal adenocarcinoma cell lines AsPC-1 (catalog no. TCHu 8), BxPC-3 (catalog no. TCHu 12), and MIA PaCa-2 (catalog no. SCSP-568) were obtained from the Cell Bank of the Chinese Academy of Sciences (Shanghai, China). Cell authentication was performed by short tandem repeat (STR) profiling within one year before the experiments, and all cell lines were confirmed to be free of mycoplasma contamination by polymerase chain reaction (PCR)-based testing. Cells were cultured in RPMI-1640 medium (MA0315; Dalian Meilun Biotechnology Co., Ltd.) supplemented with 10% fetal bovine serum (FBS; A5256701; Gibco, Thermo Fisher Scientific) at 37°C in a humidified atmosphere containing 5% CO_2_.

Cell viability was assessed using the 3-(4,5-dimethylthiazol-2-yl)-2,5-diphenyltetrazolium bromide (MTT) assay. Cells were plated in 96-well plates at a density of 5000 cells per well and incubated overnight. After attachment, cells were treated with vehicle or savolitinib at 0.5, 1, 2, 4, 6, 8, 10, or 12 μM for 24 or 48 h. The culture medium was then replaced with 100 μL of fresh medium containing 0.5 mg/mL MTT (ST316; Beyotime Biotechnology), and cells were incubated for another 4 h at 37°C. After removal of the MTT-containing medium, the formazan crystals were dissolved in 150 μL dimethyl sulfoxide (DMSO; 30072418; Sinopharm Chemical Reagent Co., Ltd., Shanghai, China). Absorbance at 570 nm was measured using an Epoch microplate spectrophotometer (BioTek Instruments, Inc., Winooski, VT, USA). Cell viability was calculated relative to the untreated control group. Each treatment condition was assessed using 4–6 technical replicate wells, as specified in the corresponding figure legends, and each MTT assay was independently repeated three times.

### 2.3 Apoptosis Analysis

Apoptosis was assessed by Annexin V-FITC/PI staining followed by flow cytometry. Cells were seeded in 6-well plates at a density of 1 × 10^5^ cells per well. For the dose-response apoptosis assay, cells were treated with savolitinib at 0, 2, 4, or 8 μM for 48 h. For the combination assay, cells were treated with savolitinib (8 μM), CQ (7.5 μM), or their combination for 48 h. After treatment, both adherent and floating cells were collected, washed with phosphate-buffered saline (PBS), and stained using the Annexin V-FITC/PI Apoptosis Detection Kit according to the manufacturer’s instructions. Flow cytometric analysis was performed using a CytoFLEX S flow cytometer (Beckman Coulter, Brea, CA, USA). Cellular debris was excluded based on forward scatter area (FSC-A) and side scatter area (SSC-A), and doublets were excluded using FSC-A versus FSC-H gating. Annexin V-FITC and PI gates were established using unstained and single-stained controls. At least 10,000 single-cell events were acquired for each sample. Viable cells (Annexin V^−^/PI^−^), early apoptotic cells (Annexin V^+^/PI^−^), late apoptotic cells (Annexin V^+^/PI^+^), and necrotic cells (Annexin V^−^/PI^+^) were distinguished and quantified using CytExpert software version 2.4 (Beckman Coulter).

### 2.4 RNA Sequencing and Bioinformatics Analysis

AsPC-1 cells were treated with 8 μM savolitinib or vehicle control for 24 h. Total RNA was extracted using TRIzol reagent (R401-01; Vazyme Biotech Co., Ltd., Nanjing, China) according to the manufacturer’s instructions. Qualified total RNA was subjected to poly(A) mRNA enrichment using oligo(dT) magnetic beads, followed by strand-specific library construction involving mRNA fragmentation, random-primed complementary DNA (cDNA) synthesis, deoxyuridine triphosphate (dUTP) incorporation during second-strand synthesis, adaptor ligation, and PCR enrichment. The libraries were sequenced on an Illumina NovaSeq platform to generate 2 × 150-bp paired-end reads by Shanghai Majorbio Bio-pharm Technology Co., Ltd. (Shanghai, China).

After quality filtering, clean reads were aligned to the human reference genome assembly GRCh38 using HISAT2 version 2.2.1, and gene-level expression was quantified as expected counts using RSEM version 1.3.3. Low-expression genes were removed before downstream analysis. Differential expression analysis was performed using DESeq2 version 1.42.0 in R, and *p*-values were adjusted using the Benjamini–Hochberg method. Genes with an adjusted *p*-value < 0.05 and |log_2_ fold change| ≥ 1 were defined as high-confidence differentially expressed genes. Variance-stabilizing transformed (VST) values were used for principal component analysis and visualization. Gene set enrichment analysis was performed using genes ranked by the DESeq2 Wald statistic.

### 2.5 Western Blot

After the indicated treatments, cells were washed with cold phosphate-buffered saline (PBS) and lysed in radioimmunoprecipitation assay (RIPA) lysis buffer (P0013B; Beyotime Biotechnology) to extract total protein. Protein concentration was measured using a bicinchoninic acid (BCA) protein assay kit (P0010; Beyotime Biotechnology) according to the manufacturer’s instructions. Protein samples were mixed with loading buffer (P0286; Beyotime Biotechnology) and denatured at 100°C for 5 min. Approximately 20–30 μg of total protein per lane was separated on 8–15% sodium dodecyl sulfate–polyacrylamide gel electrophoresis (SDS-PAGE) gels, with the gel percentage selected according to the molecular weight of the target protein, and subsequently transferred onto 0.22 μm polyvinylidene fluoride (PVDF) membranes (ISEQ00005; Merck Millipore, Burlington, MA, USA). After blocking with 5% bovine serum albumin (BSA; MB4219; Meilun Biotechnology Co., Ltd., Dalian, China) for 2 h at room temperature, the membranes were incubated with the indicated primary antibodies overnight at 4°C. After washing with Tris-buffered saline containing Tween 20 (TBST), the membranes were incubated with HRP-conjugated secondary antibodies (RA1008; Vazyme Biotech Co., Ltd.; 1:5000) for 2 h at room temperature. Protein bands were visualized using enhanced chemiluminescence reagents (34580; Thermo Fisher Scientific) and imaged with a ChemiDoc MP Imaging System (Bio-Rad Laboratories, Hercules, CA, USA). Band intensities were quantified using ImageJ software version 1.53t (National Institutes of Health, Bethesda, MD, USA).

### 2.6 Confocal Microscopy and Transmission Electron Microscopy

Cells were seeded in glass-bottom dishes and treated with 8 μM savolitinib for 24 h for autophagosome visualization. Rapamycin (100 nM; S115842; Shanghai Aladdin Biochemical Technology Co., Ltd., Shanghai, China) was used as a positive control for autophagy induction. For the time-course analysis of autophagic flux, cells were treated with 8 μM savolitinib for 12, 24, or 48 h. After treatment, cells were simultaneously stained at 37°C for 30 min in serum-free culture medium containing Hoechst 33342 and MDC at 1:1000 dilutions and LysoTracker Red DND-99 at a 1:5000 dilution. The cells were then washed twice with phosphate-buffered saline (PBS) and imaged in PBS using an LSM 710 confocal laser scanning microscope (Carl Zeiss, Jena, Germany).

For transmission electron microscopy (TEM) analysis, cells were treated with 8 μM savolitinib for 24 h, harvested by centrifugation at 300× *g* for 5 min at 4°C, and fixed with electron microscopy fixative (G1102; Servicebio Technology Co., Ltd., Wuhan, China). Sample processing, ultrathin sectioning, and TEM imaging were performed by Servicebio Technology Co., Ltd. Autophagosome-like double-membrane vesicles and autolysosome-like structures were observed and recorded.

### 2.7 Tumor Xenograft Model

The animal experimental protocol was approved by the Animal Ethical Committee of the School of Pharmacy of Fudan University (Shanghai, China; approval No. 2025-01-SY-ZXY-02). A total of 30 female NCG (NOD/ShiLtJGpt-Prkdc^em26Cd52^Il2rg^em26Cd22^/Gpt) immunodeficient mice (6–8 weeks old, weighing 18–20 g; GemPharmatech Co., Ltd., Nanjing, China) were maintained under specific pathogen-free (SPF) conditions. Female NCG immunodeficient mice were selected because their relatively low level of aggressive behavior facilitates group housing and longitudinal monitoring during xenograft experiments. Mice of the same sex and age range were used across all treatment groups to maintain experimental consistency.

Subcutaneous xenografts were established by injecting 5 × 10^6^ BxPC-3 cells suspended in 100 μL phosphate-buffered saline (PBS) into the flank of each mouse. When the average tumor volume reached approximately 200 mm^3^, mice were randomly assigned to five groups (n = 6 per group): vehicle control, savolitinib, chloroquine, savolitinib plus chloroquine, and gemcitabine. Savolitinib was administered by oral gavage at 50 mg/kg once daily, chloroquine was administered intraperitoneally at 40 mg/kg once daily, and gemcitabine was administered intravenously at 50 mg/kg every two days. The vehicle control group received the corresponding vehicle according to the same schedule.

Tumor size and body weight were measured twice weekly, and tumor volume was calculated as length × width^2^/2. At the end of treatment, mice were euthanized, and tumors and major organs were collected for subsequent analyses. For euthanasia, mice received an overdose of sodium pentobarbital (150 mg/kg) by intraperitoneal injection. Humane endpoints were predefined clinical or tumor-burden criteria requiring immediate euthanasia to prevent unnecessary suffering. These endpoints included severe lethargy, inability to access food or water, body weight loss exceeding 20%, tumor ulceration or impaired mobility due to tumor burden, or tumor volume exceeding 1500 mm^3^. Animal welfare was monitored daily throughout the study. Tumor growth inhibition was calculated at the study endpoint using the following formula: TGI (%) = [1 − (mean tumor volume of the treatment group/mean tumor volume of the vehicle control group)] × 100%.

### 2.8 Immunohistochemistry

Tumor tissues and major organs were fixed in 4% paraformaldehyde (PFA), embedded in paraffin, and sectioned at 4 μm. For histological analysis, sections were subjected to H&E staining. For immunohistochemistry (IHC), tumor sections were deparaffinized and rehydrated, followed by heat-induced antigen retrieval in ethylenediaminetetraacetic acid (EDTA) buffer (pH 9.0) using a microwave oven for 20 min. Endogenous peroxidase activity was quenched with 3% hydrogen peroxide for 25 min, and nonspecific binding was blocked with 3% BSA (MB4219; Meilun Biotechnology Co., Ltd.) for 30 min at room temperature. The sections were then incubated overnight at 4°C with primary antibodies against Ki-67 (GB121499-100; Servicebio Technology Co., Ltd.), cleaved caspase-3 (9661; Cell Signaling Technology), and PCNA (GB11010; Servicebio Technology Co., Ltd.). After washing, the sections were incubated with the corresponding HRP-conjugated secondary antibody (GB23301; Servicebio Technology Co., Ltd., Wuhan, China; 1:500) for 1 h at room temperature. The sections were developed with DAB for 5 min and counterstained with hematoxylin. All slides were scanned using a VS200 whole-slide scanner (Olympus, Tokyo, Japan) for subsequent analysis.

### 2.9 Statistical Analysis

Quantitative data are presented as the mean ± standard deviation (SD). Statistical analyses were performed using OriginPro (version 2024; OriginLab Corporation, Northampton, MA, USA). Comparisons between two groups were performed using an unpaired two-tailed Student’s *t*-test. For comparisons among multiple groups, one-way analysis of variance (ANOVA) followed by Tukey’s *post hoc* test was used. Differences were considered statistically significant at *p* < 0.05. Longitudinal tumor-volume and body-weight data were analyzed using two-way repeated-measures ANOVA. Significance levels are indicated as follows: *, *p* < 0.05; **, *p* < 0.01; ***, *p* < 0.001; and ****, *p* < 0.0001.

## 3 Results

### 3.1 Savolitinib Suppresses MET Signaling and Cell Viability in MET-High Pancreatic Cancer Cells

**Figure 1:**
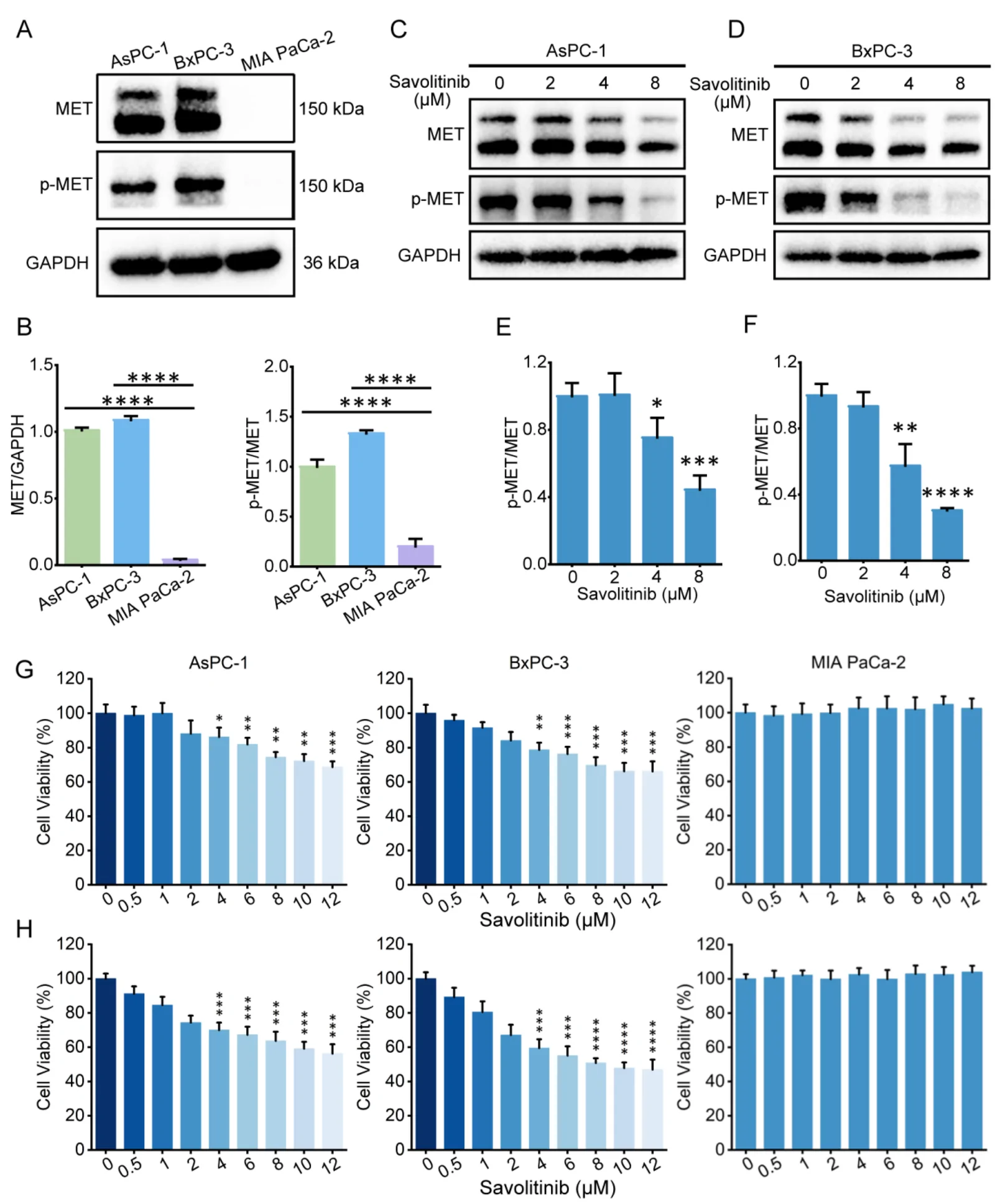
**Savolitinib suppresses MET phosphorylation and reduces cell viability in MET-high pancreatic cancer cells.** (**A**) Representative western blot images showing basal MET and p-MET levels in AsPC-1, BxPC-3, and MIA PaCa-2 cells. (**B**) Densitometric quantification of total MET normalized to GAPDH and p-MET normalized to total MET. MIA PaCa-2 cells were used as the reference group for statistical comparisons. (n = 3). (**C**,**D**) Representative western blot images of MET and p-MET in AsPC-1 (**C**) and BxPC-3 (**D**) cells after treatment with the indicated concentrations of savolitinib for 48 h. (**E**,**F**) Densitometric quantification of the p-MET/total MET ratio in AsPC-1 (**E**) and BxPC-3 (**F**) cells. (**G**) Cell viability of AsPC-1, BxPC-3, and MIA PaCa-2 cells after savolitinib treatment for 24 h, as determined by the MTT assay (n = 5). (**H**) Cell viability of AsPC-1, BxPC-3, and MIA PaCa-2 cells after savolitinib treatment for 48 h, as determined by the MTT assay (n = 5). Data are presented as the mean ± SD. Statistical significance was determined by one-way ANOVA followed by Tukey’s *post hoc* test. Unless otherwise indicated, comparisons were made with the corresponding control group. *, *p* < 0.05; **, *p* < 0.01; ***, *p* < 0.001; ****, *p* < 0.0001.

Savolitinib is a selective MET tyrosine kinase inhibitor [9]; therefore, inhibition of MET phosphorylation was used as the primary pharmacodynamic readout of MET pathway inhibition. To evaluate the antitumor activity of savolitinib in pancreatic cancer cells, we first examined basal MET expression and activation in three pancreatic cancer cell lines. Western blot analysis showed that AsPC-1 and BxPC-3 cells exhibited substantially higher total MET expression than MIA PaCa-2 cells. Quantification of the p-MET/total MET ratio further indicated elevated basal MET phosphorylation in AsPC-1 and BxPC-3 cells compared with MIA PaCa-2 cells (Fig. 1A,B).

We next assessed whether savolitinib effectively inhibited MET activation in MET-high pancreatic cancer cells. Quantification of the p-MET/total MET ratio showed that savolitinib markedly suppressed MET phosphorylation in AsPC-1 and BxPC-3 cells, particularly at concentrations of 4 and 8 μM after 48 h of treatment (Fig. 1C–F). Total MET protein levels normalized to GAPDH also decreased following savolitinib treatment.

The effect of savolitinib on cell viability was then evaluated using the MTT assay. Savolitinib significantly reduced the viability of MET-high AsPC-1 and BxPC-3 cells in a dose- and time-dependent manner (Fig. 1G,H). In contrast, MET-low MIA PaCa-2 cells were less sensitive to savolitinib treatment under the same conditions. These findings suggest that the growth-inhibitory effect of savolitinib is associated with basal MET expression and MET pathway activity in pancreatic cancer cells.

### 3.2 Savolitinib Induces Apoptosis in MET-High Pancreatic Cancer Cells

**Figure 2:**
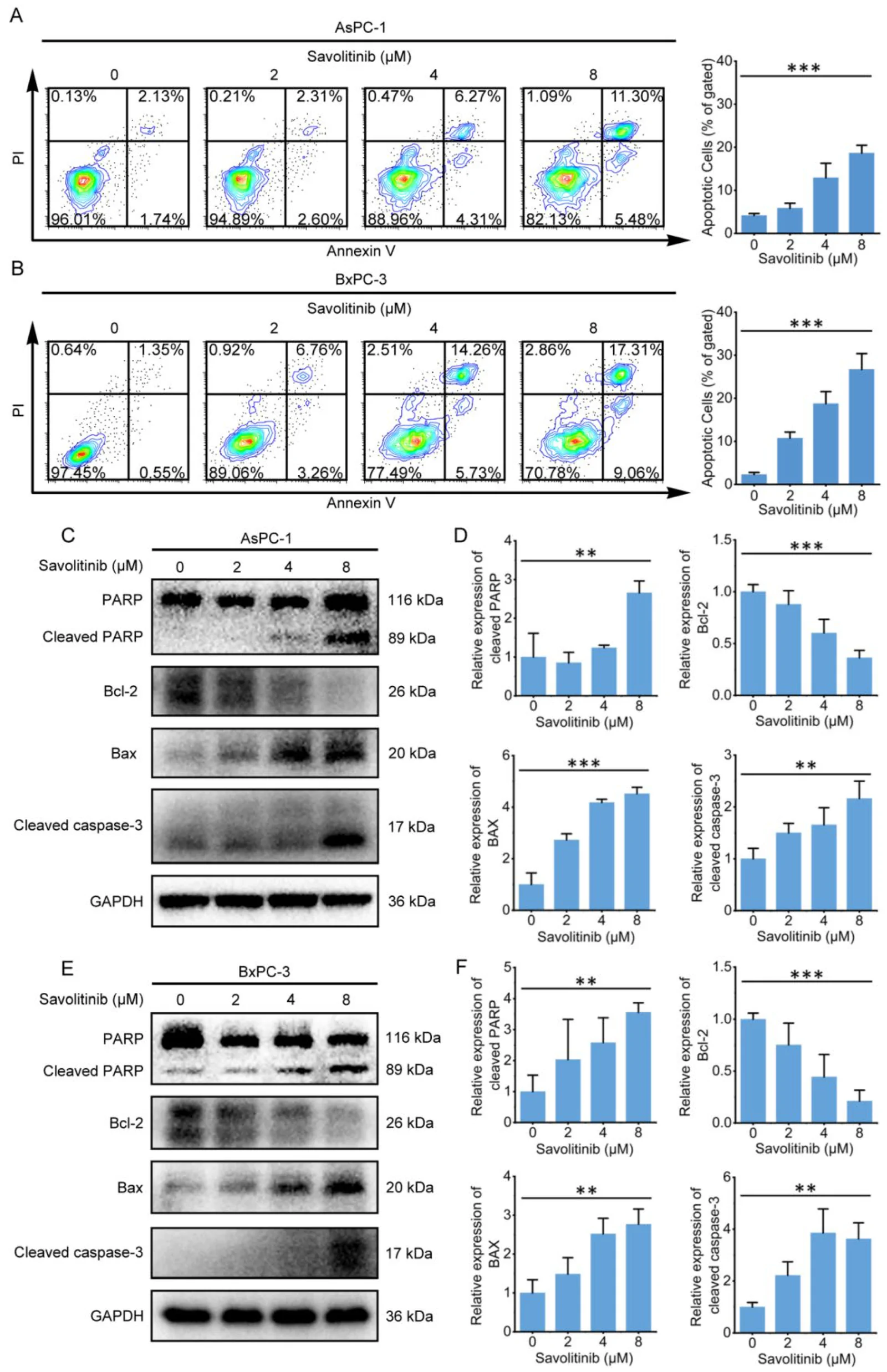
**Savolitinib induces apoptosis in MET-high pancreatic cancer cells.** (**A**) Apoptotic rate of AsPC-1 cells after treatment with the indicated concentrations of savolitinib for 48 h, as determined by Annexin V-FITC/PI staining followed by flow cytometry (n = 3). (**B**) Apoptotic rate of BxPC-3 cells after treatment with the indicated concentrations of savolitinib for 48 h (n = 3). (**C**) Representative western blot images of apoptosis-related proteins in AsPC-1 cells after savolitinib treatment for 48 h. (**D**) Quantification of cleaved PARP, Bcl-2, Bax, and cleaved caspase-3 protein levels in AsPC-1 cells (n = 3). (**E**) Representative western blot images of apoptosis-related proteins in BxPC-3 cells after savolitinib treatment for 48 h. (**F**) Quantification of cleaved PARP, Bcl-2, Bax, and cleaved caspase-3 protein levels in BxPC-3 cells (n = 3). Data are presented as the mean ± SD. Statistical significance was determined by one-way ANOVA followed by Tukey’s *post hoc* test. Comparisons were made with the untreated control group. **, *p* < 0.01; ***, *p* < 0.001.

To elucidate the mechanism of savolitinib-induced cell death, we performed Annexin V-FITC/PI staining followed by flow cytometry analysis. Treatment with savolitinib resulted in a significant and dose-dependent increase in the percentage of both early (Annexin V^+^/PI^-^) and late (Annexin V^+^/PI^+^) apoptotic cells in AsPC-1 and BxPC-3 cells after 48 h of treatment (Fig. 2A,B). At 8 μM savolitinib, the total apoptotic population increased from 4.15 ± 0.46% in the control group to 18.66 ± 1.80% in AsPC-1 cells, and from 2.29 ± 0.48% to 26.71 ± 3.66% in BxPC-3 cells. In contrast, the MET-low MIA PaCa-2 cell line showed no obvious increase in apoptosis under the same treatment conditions (Fig. S1), suggesting that MET-high pancreatic cancer cells were more susceptible to savolitinib-induced apoptosis.

We next examined the expression of apoptosis-related proteins by Western blotting. Consistent with the flow cytometry results, savolitinib increased the levels of cleaved PARP and cleaved caspase-3 in AsPC-1 and BxPC-3 cells. In addition, savolitinib treatment increased the expression of the pro-apoptotic protein Bax and decreased the expression of the anti-apoptotic protein Bcl-2, resulting in an increased Bax/Bcl-2 ratio (Fig. 2C–F). These changes indicate activation of apoptotic signaling in response to savolitinib treatment.

Taken together, these results show that savolitinib induces apoptosis in MET-high pancreatic cancer cells, which contributes to its growth-inhibitory effects.

### 3.3 Transcriptomic Analysis Reveals Activation of Autophagy- and Stress-Related Programs Following Savolitinib Treatment

**Figure 3:**
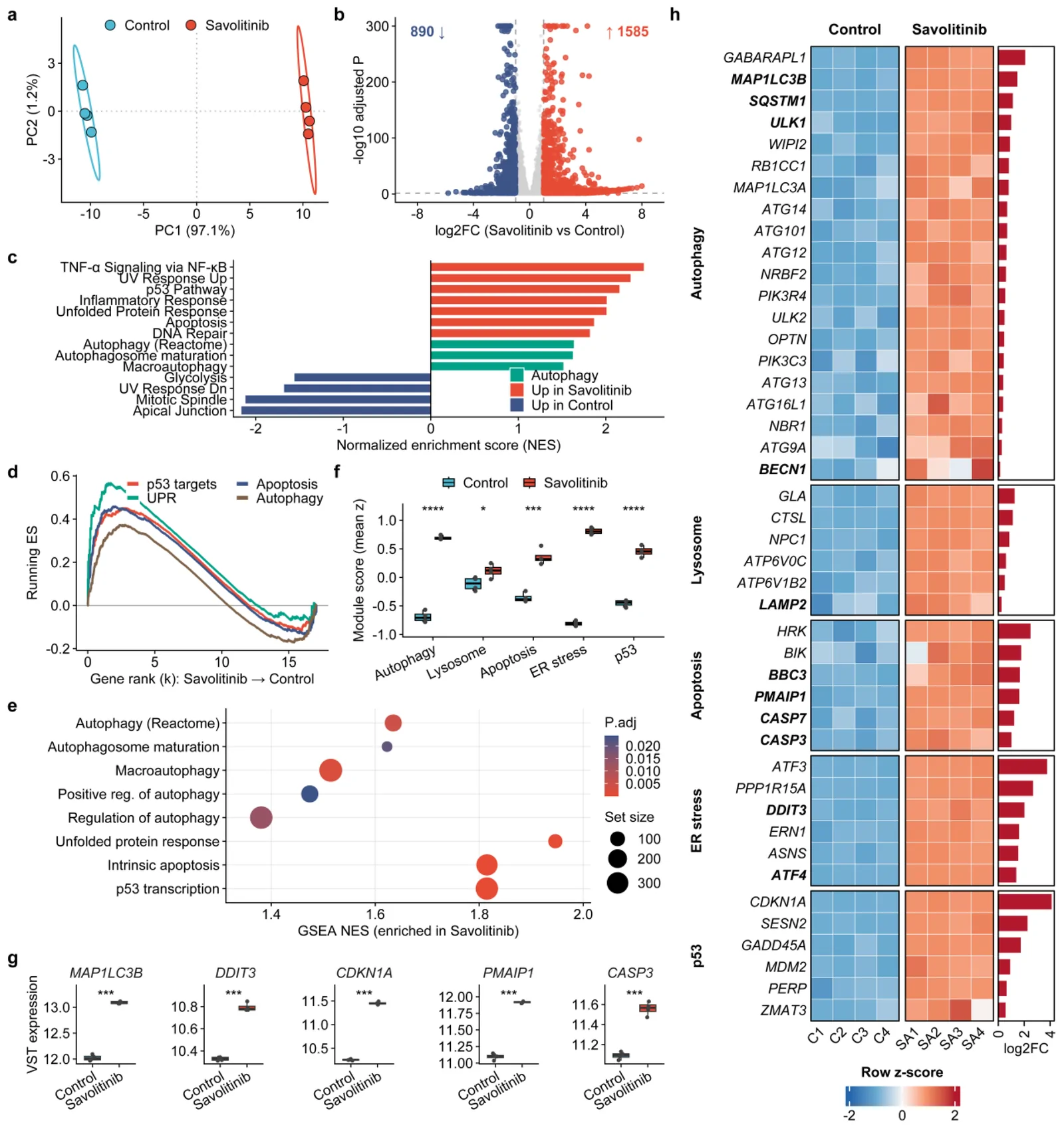
**Transcriptomic analysis reveals activation of autophagy- and stress-related programs after savolitinib treatment.** (**a**) Principal component analysis (PCA) based on variance-stabilizing transformed (VST) expression values of the 2000 most variable genes. (**b**) Volcano plot of differentially expressed genes after savolitinib treatment. Red and blue dots indicate upregulated and downregulated genes, respectively, defined by adjusted *p* < 0.05 and |log_2_FC| ≥ 1. (**c**) Gene set enrichment analysis (GSEA) showing normalized enrichment scores of representative pathways. (**d**) GSEA enrichment curves for p53 targets, intrinsic apoptosis, unfolded protein response (UPR), and macroautophagy gene sets. (**e**) GSEA dot plot of autophagy-related and stress- or cell death-related gene sets. (**f**) Module scores for autophagy, lysosome, apoptosis, endoplasmic reticulum (ER) stress, and p53-related gene programs. (**g**) VST expression levels of selected representative genes. (**h**) Heatmap showing representative genes related to autophagy, lysosomal function, apoptosis, ER stress, and p53 signaling. Colors indicate row-scaled VST expression z-scores, and the bars on the right indicate log2 fold changes. Data are from four biological replicates per group. Statistical significance in (**f**) was determined by unpaired two-tailed Student’s *t*-test, and statistical significance in (**g**) was based on DESeq2 analysis. *, *p* < 0.05; ***, *p* < 0.001; ****, *p* < 0.0001.

To explore the transcriptional changes induced by savolitinib, RNA sequencing was performed in AsPC-1 cells treated with savolitinib or vehicle control for 24 h. Principal component analysis showed a clear separation between the control and savolitinib-treated groups, with tight clustering of biological replicates within each group, indicating a reproducible transcriptional response to savolitinib treatment (Fig. 3a). Differential expression analysis identified 2475 high-confidence differentially expressed genes, including 1585 upregulated and 890 downregulated genes, using an adjusted *p*-value < 0.05 and |log_2_FC| ≥ 1 as thresholds (Fig. 3b).

Gene set enrichment analysis further showed that savolitinib treatment was associated with activation of stress- and cell death-related transcriptional programs, including p53 signaling, unfolded protein response, and apoptosis (Fig. 3c,d). Notably, autophagy-related gene sets, including macroautophagy, autophagosome maturation, and Reactome autophagy, were also enriched in the savolitinib-treated group (Fig. 3c–e). Consistently, pathway module analysis showed increased module scores for autophagy, lysosome, apoptosis, endoplasmic reticulum stress, and p53-related programs after savolitinib treatment (Fig. 3f). Representative genes related to these processes showed concordant transcriptional changes (Fig. 3g), and heatmap analysis further illustrated the upregulation of selected genes involved in autophagy, lysosomal function, apoptosis, endoplasmic reticulum stress, and p53 signaling (Fig. 3h).

Together, these transcriptomic data suggest that savolitinib induces a coordinated transcriptional response involving autophagy-lysosome-related transcriptional programs, cellular stress, and apoptotic signaling in MET-high pancreatic cancer cells. These findings prompted us to further examine whether savolitinib induces autophagic activity and autophagic flux at the cellular and protein levels.

### 3.4 Savolitinib Induces Autophagy-Associated Changes and Suppresses AKT/mTOR Signaling in MET-High Pancreatic Cancer Cells

**Figure 4:**
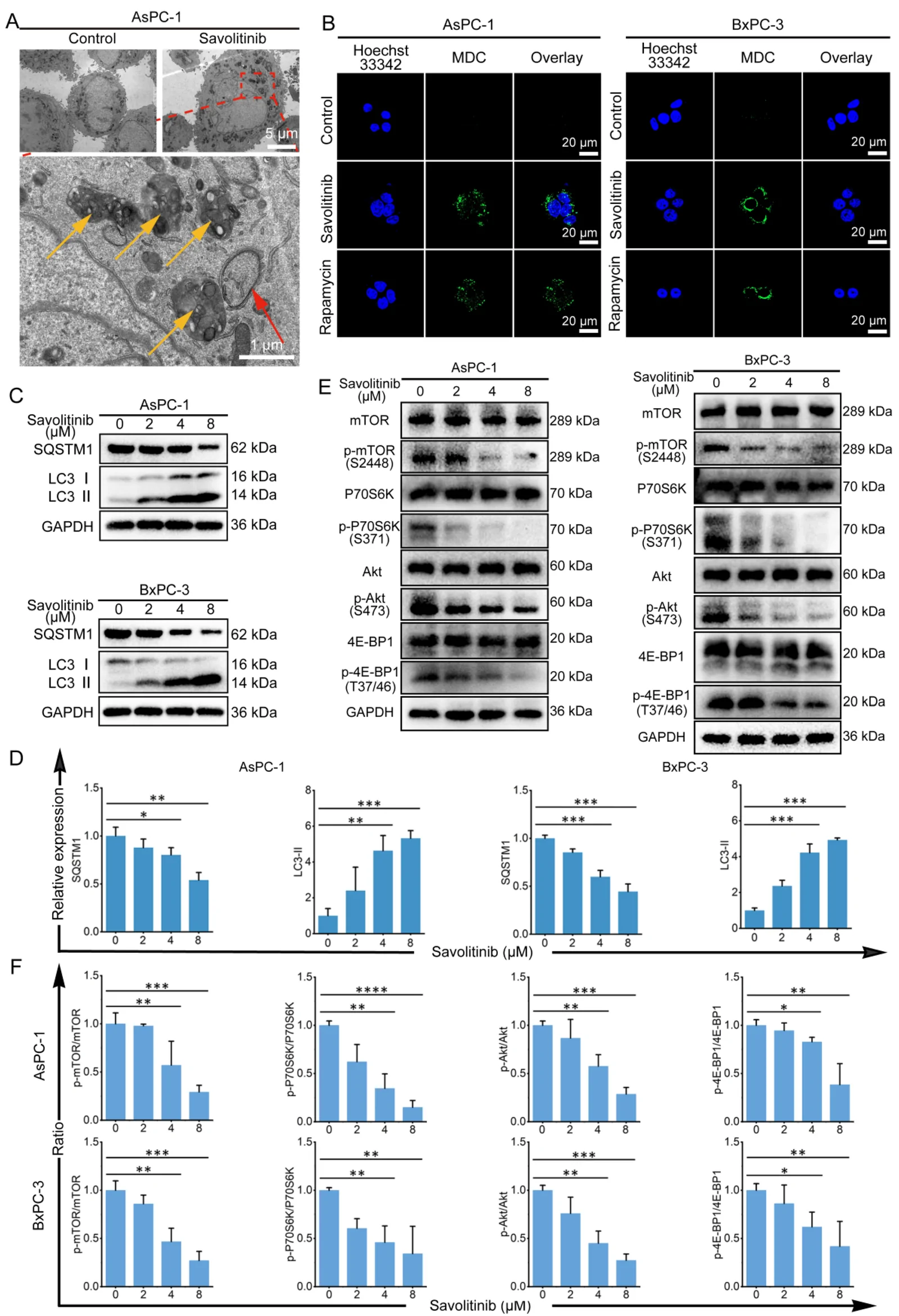
**Savolitinib induces autophagy-associated changes and suppresses AKT/mTOR signaling in pancreatic cancer cells.** (**A**) Representative transmission electron microscopy images of AsPC-1 cells treated with vehicle or savolitinib for 24 h. A putative autophagosome-like double-membrane vesicle is indicated by a red arrow, whereas autolysosome-like degradative vacuoles are indicated by orange arrowheads. Scale bars, 5 μm in the upper images and 1 μm in the lower image. (**B**) Representative fluorescence images of MDC staining in AsPC-1 and BxPC-3 cells after treatment with vehicle or savolitinib for 24 h. Scale bars, 20 μm. (**C**) Representative western blots of autophagy-related proteins in AsPC-1 and BxPC-3 cells after treatment with the indicated concentrations of savolitinib for 24 h. (**D**) Quantification of the autophagy-related protein levels shown in (**C**) (n = 3). (**E**) Representative western blots of phosphorylated and total AKT, mTOR, p70S6K, and 4E-BP1 in AsPC-1 and BxPC-3 cells after treatment with the indicated concentrations of savolitinib for 24 h. (**F**) Quantification of the p-AKT/AKT, p-mTOR/mTOR, p-p70S6K/p70S6K, and p-4E-BP1/4E-BP1 ratios shown in (**E**) (n = 3). Data are presented as the mean ± SD. Statistical significance was determined by one-way ANOVA followed by Tukey’s *post hoc* test. Comparisons were made with the untreated control group. *, *p* < 0.05; **, *p* < 0.01; ***, *p* < 0.001; ****, *p* < 0.0001.

To validate the transcriptomic findings at the morphological level, transmission electron microscopy was performed to examine the ultrastructural changes induced by savolitinib. Compared with vehicle-treated AsPC-1 cells, savolitinib-treated cells displayed a double-membrane vesicle containing cytoplasmic material, morphologically consistent with an autophagosome-like structure, together with several electron-dense degradative vacuoles resembling autolysosome-like structures (Fig. 4A). In parallel, MDC staining revealed increased punctate fluorescence in both AsPC-1 and BxPC-3 cells after savolitinib treatment. Rapamycin, an mTOR inhibitor commonly used to induce autophagy, was included as a positive control and produced a similar increase in MDC-positive puncta (Fig. 4B) [17]. These morphological observations support the induction of an autophagy-associated response by savolitinib in MET-high pancreatic cancer cells.

Western blot analysis was subsequently performed to examine changes in autophagy-related proteins. Savolitinib treatment increased LC3-II accumulation in a dose-dependent manner in both AsPC-1 and BxPC-3 cells, while reducing SQSTM1/p62 protein levels (Fig. 4C,D). Although SQSTM1 was transcriptionally upregulated in the RNA-seq analysis, its protein level decreased after savolitinib treatment, indicating discordant regulation at the transcript and protein levels and suggesting increased post-translational turnover of SQSTM1/p62. Because steady-state LC3-II and SQSTM1/p62 levels alone cannot fully distinguish increased autophagosome formation from altered lysosomal degradation, autophagic flux was further examined in subsequent experiments [18]. Together with the morphological findings, these protein-level changes support enhanced autophagic activity following savolitinib treatment.

We next examined the AKT/mTOR signaling pathway, a major negative regulator of autophagy [17]. Savolitinib treatment dose-dependently reduced the p-AKT/AKT, p-mTOR/mTOR, p-p70S6K/p70S6K, and p-4E-BP1/4E-BP1 ratios in both AsPC-1 and BxPC-3 cells, whereas the corresponding total protein levels remained relatively unchanged (Fig. 4E,F). These findings indicate suppression of AKT/mTOR pathway activity and suggest that inhibition of this pathway may contribute to the autophagy-associated response induced by MET inhibition.

Collectively, these findings show that savolitinib induces a coordinated autophagy-associated response in MET-high pancreatic cancer cells, characterized by increased autophagy-related vesicular structures, LC3-II accumulation, and reduced SQSTM1/p62 protein levels. The concurrent suppression of AKT/mTOR signaling further suggests that this pathway may be involved in savolitinib-induced autophagic activity.

### 3.5 Savolitinib Promotes Autophagic Activity and Autophagic Flux in MET-High Pancreatic Cancer Cells

**Figure 5:**
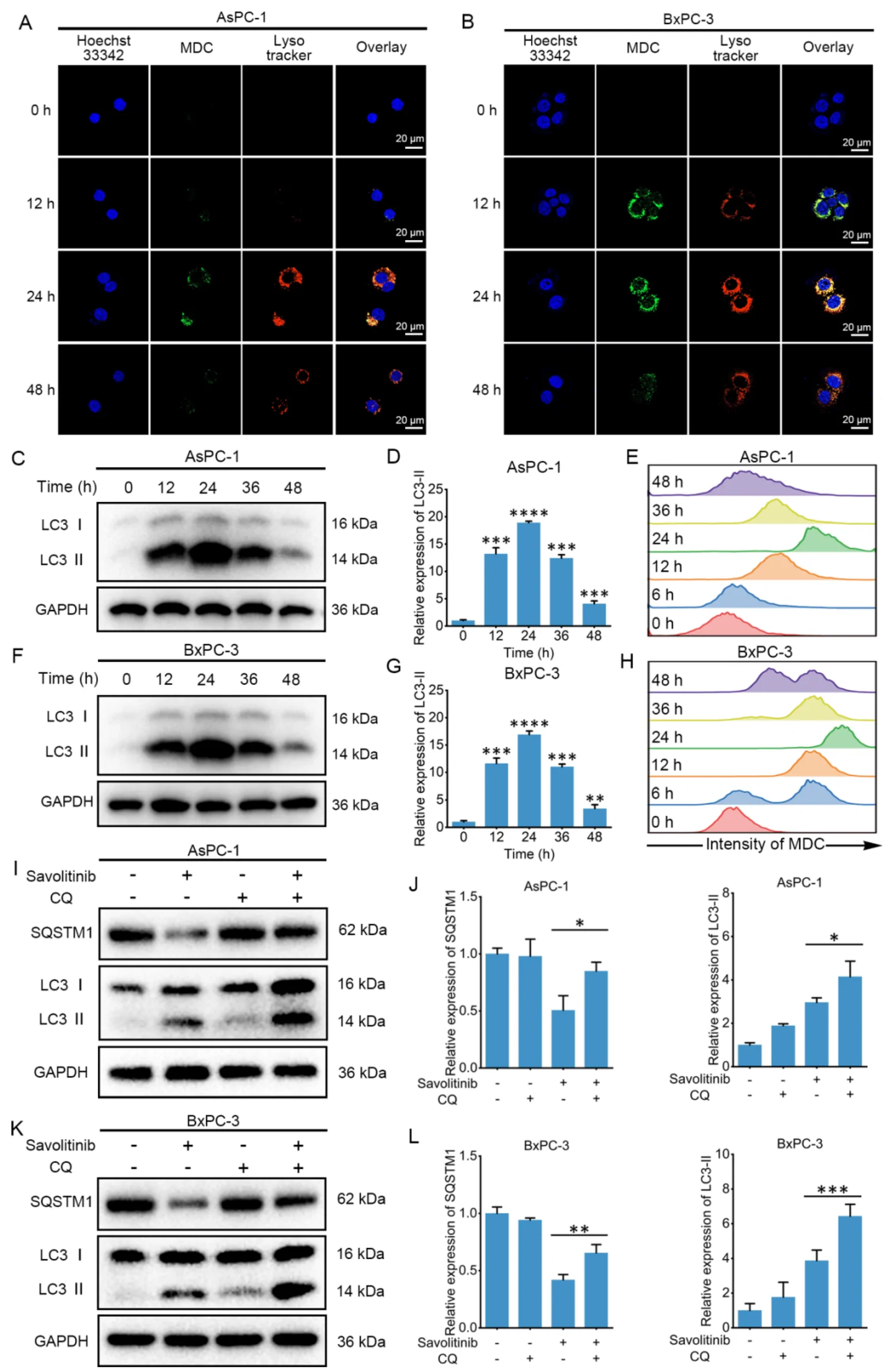
**Savolitinib induces autophagic flux in pancreatic cancer cells.** (**A**) Confocal images of AsPC-1 cells stained with MDC and LysoTracker Red DND-99 after savolitinib treatment for the indicated durations. (**B**) Confocal images of BxPC-3 cells stained as described in (**A**). MDC-positive puncta are shown in green, LysoTracker-positive lysosomes are shown in red, and yellow puncta indicate potential co-localization. Scale bars, 20 μm. (**C**) Representative LC3 western blots in AsPC-1 cells after savolitinib treatment for the indicated durations. (**D**) Quantification of LC3-II levels in AsPC-1 cells is shown in (**C**) (n = 4). (**E**) Representative flow cytometry histograms of MDC fluorescence intensity in AsPC-1 cells after savolitinib treatment. (**F**) Representative LC3 western blots in BxPC-3 cells after savolitinib treatment for the indicated durations. (**G**) Quantification of LC3-II levels in BxPC-3 cells shown in (**F**) (n = 4). (**H**) Representative flow cytometry histograms of MDC fluorescence in BxPC-3 cells after savolitinib treatment. (**I**) Representative western blots of SQSTM1/p62 and LC3 in AsPC-1 cells treated with savolitinib, chloroquine (CQ), or their combination for 24 h. (**J**) Quantification of SQSTM1/p62 and LC3-II levels in AsPC-1 cells shown in (**I**) (n = 3). (**K**) Representative western blots of SQSTM1/p62 and LC3 in BxPC-3 cells treated with savolitinib, CQ, or their combination for 24 h. (**L**) Quantification of SQSTM1/p62 and LC3-II levels in BxPC-3 cells shown in (**K**) (n = 3). Data are presented as the mean ± SD. Statistical significance was determined by one-way ANOVA followed by Tukey’s *post hoc* test. Unless otherwise indicated, comparisons were made with the corresponding untreated or 0 h control group. *, *p* < 0.05; **, *p* < 0.01; ***, *p* < 0.001; ****, *p* < 0.0001.

To visualize temporal changes in autophagy-associated vesicular structures, AsPC-1 and BxPC-3 cells were stained with MDC and LysoTracker Red DND-99 after savolitinib treatment for different durations. Compared with untreated cells, savolitinib-treated cells showed increased MDC-positive puncta, which became more evident at 12–24 h. Partial co-localization between MDC-positive puncta and LysoTracker-positive lysosomes was also observed in the merged images, suggesting increased association between autophagic vacuole-like structures and lysosomal compartments (Fig. 5A,B). At later time points, the MDC signal declined, whereas LysoTracker-positive vesicles remained detectable. These time-dependent changes suggested that savolitinib enhanced autophagy-associated vesicle formation and lysosomal involvement in MET-high pancreatic cancer cells.

To further characterize the temporal dynamics of this autophagic response, LC3-II levels were examined by Western blotting. Because steady-state LC3-II levels reflect the balance between autophagosome formation and subsequent lysosomal turnover, LC3-II changes should be interpreted in a time-dependent manner and in combination with flux inhibition assays [18]. In both AsPC-1 and BxPC-3 cells, savolitinib markedly increased LC3-II levels, with the strongest accumulation observed at 12–24 h, followed by a decline at 48 h (Fig. 5C,D,F,G). Consistently, flow cytometric analysis of MDC-stained cells showed increased MDC fluorescence intensity during the early-to-middle phase of treatment, followed by a reduction at later time points (Fig. 5E,H). Together with the confocal imaging results, these findings indicate that savolitinib induces a dynamic autophagy-associated response in MET-high pancreatic cancer cells.

To determine whether these changes reflected increased autophagic flux, we used chloroquine (CQ), a late-stage autophagy inhibitor that interferes with autophagosome-lysosome fusion and lysosome-dependent turnover [18,19]. In AsPC-1 and BxPC-3 cells, savolitinib alone increased LC3-II levels and reduced SQSTM1/p62 expression, suggesting enhanced autophagosome formation accompanied by substrate turnover. Compared with savolitinib treatment alone, co-treatment with CQ led to further accumulation of LC3-II and prevented the reduction of SQSTM1/p62 (Fig. 5I–L). These results indicate that savolitinib promotes autophagosome generation and autophagic substrate turnover rather than simply blocking late-stage autophagy, thereby supporting the induction of autophagic flux in MET-high pancreatic cancer cells.

Collectively, these results indicate that savolitinib induces a dynamic autophagic response in MET-high pancreatic cancer cells, characterized by early-to-middle phase accumulation of autophagy-associated vesicular structures and LC3-II, followed by subsequent turnover. The further accumulation of LC3-II and prevention of SQSTM1/p62 reduction after CQ co-treatment further support that savolitinib promotes autophagic flux rather than simply blocking late-stage autophagy.

### 3.6 Autophagy Inhibition Enhances Savolitinib-Induced Growth Inhibition and Apoptosis

After observing that savolitinib induced autophagic flux, we next investigated the functional role of this autophagic response. Autophagy can either promote cancer cell survival or contribute to cell death under anticancer treatment, depending on the cellular context and therapeutic stress [15]. To determine whether savolitinib-induced autophagy functioned as a pro-survival or pro-death response, we pharmacologically modulated autophagy in combination with savolitinib. CQ was used to inhibit late-stage autophagic flux, whereas rapamycin was used to further activate autophagy through mTOR inhibition.

**Figure 6:**
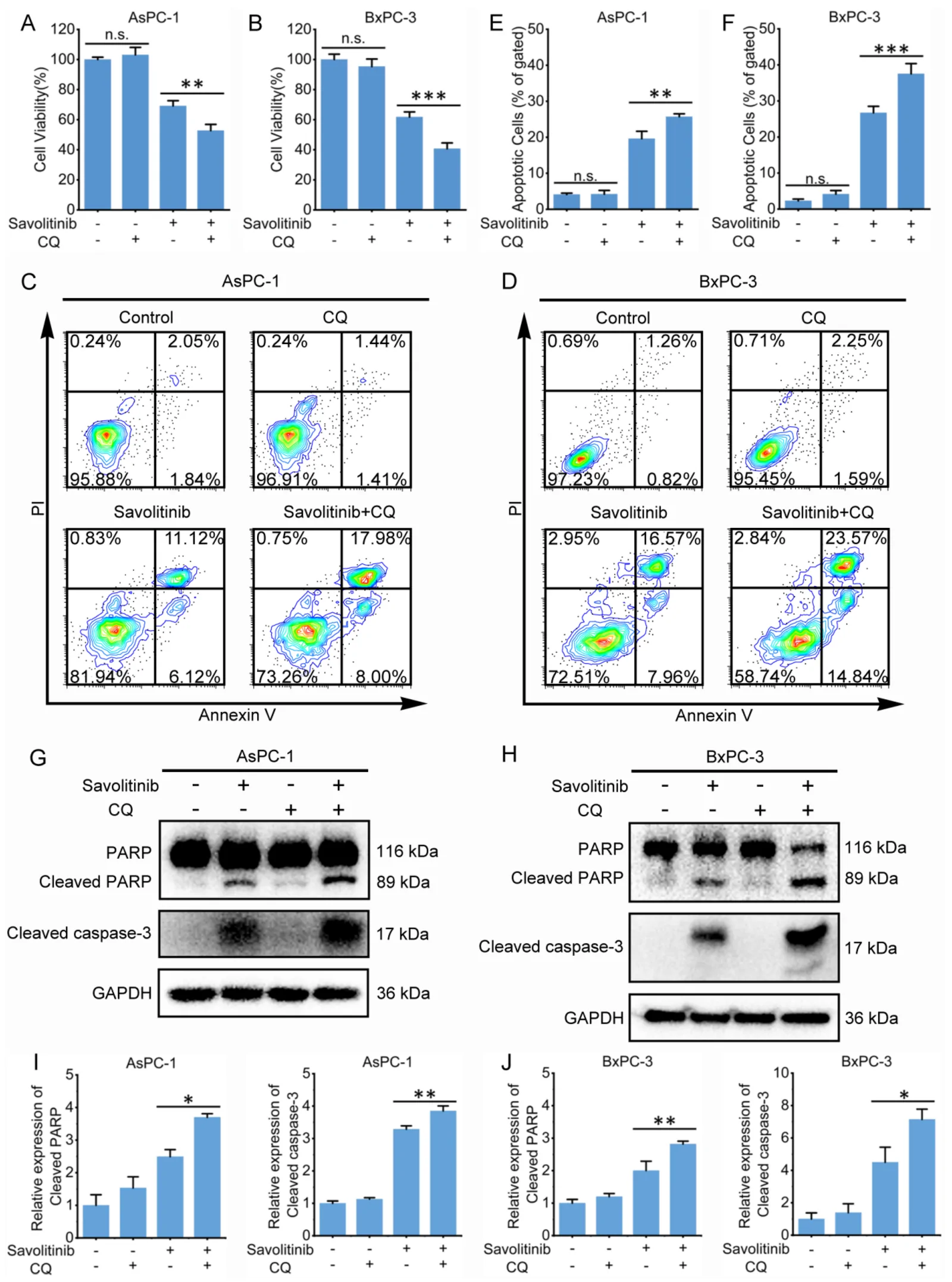
**Autophagy inhibition enhances savolitinib-induced growth inhibition and apoptosis in pancreatic cancer cells.** (**A**) Cell viability of AsPC-1 cells after treatment with savolitinib (8 μM), chloroquine (CQ; 7.5 μM), or their combination for 48 h, as determined by the MTT assay (n = 4). (**B**) Cell viability of BxPC-3 cells after the same treatments for 48 h (n = 4). (**C**) Representative flow cytometry plots of AsPC-1 cells after the indicated treatments for 48 h. (**D**) Representative flow cytometry plots of BxPC-3 cells after the indicated treatments for 48 h. (**E**) Quantification of apoptotic AsPC-1 cells is shown in (**C**) (n = 3). (**F**) Quantification of apoptotic BxPC-3 cells is shown in (**D**) (n = 3). (**G**) Representative western blots of cleaved PARP and cleaved caspase-3 in AsPC-1 cells after the indicated treatments for 48 h. (**H**) Representative western blots of cleaved PARP and cleaved caspase-3 in BxPC-3 cells after the indicated treatments for 48 h. (**I**) Quantification of apoptosis-related protein levels in AsPC-1 cells shown in (**G**) (n = 3). (**J**) Quantification of apoptosis-related protein levels in BxPC-3 cells shown in (**H**) (n = 3). Data are presented as the mean ± SD. Statistical significance was determined by one-way ANOVA followed by Tukey’s *post hoc* test. n.s., not significant; *, *p* < 0.05; **, *p* < 0.01; ***, *p* < 0.001.

We first selected concentrations of CQ and rapamycin that had minimal effects on cell viability when used alone. MTT assays showed that CQ at 7.5 μM and rapamycin at 60 nM caused only limited viability reduction in AsPC-1 and BxPC-3 cells (Fig. S2). We then combined these autophagy modulators with savolitinib to assess whether altering autophagy affected the cellular response to MET inhibition. CQ co-treatment significantly enhanced the savolitinib-induced reduction in cell viability in both AsPC-1 and BxPC-3 cells (Fig. 6A,B). In contrast, rapamycin co-treatment partially attenuated the growth-inhibitory effect of savolitinib (Fig. S3). Thus, the bidirectional modulation of autophagy produced opposite effects on savolitinib sensitivity, supporting the interpretation that autophagy induced by savolitinib predominantly functions as an adaptive cytoprotective response rather than a cytotoxic mechanism.

The effect of CQ co-treatment on savolitinib-induced apoptosis was further evaluated by Annexin V-FITC/PI staining. Flow cytometric analysis showed that the combination of savolitinib and CQ markedly increased the proportion of apoptotic cells compared with either treatment alone in both AsPC-1 and BxPC-3 cells (Fig. 6C–F). Consistently, Western blotting showed higher levels of cleaved PARP and cleaved caspase-3 in the combination group than in the single-treatment groups (Fig. 6G–J). These results indicate that inhibition of autophagy with CQ enhances savolitinib-induced apoptotic cell death in MET-high pancreatic cancer cells.

Together, these complementary pharmacological interventions support a cytoprotective role for savolitinib-induced autophagy in MET-high pancreatic cancer cells. Inhibition of autophagic flux with CQ enhanced savolitinib-induced growth inhibition and apoptosis, whereas further activation of autophagy with rapamycin partially attenuated the cytotoxic effect of savolitinib.

### 3.7 Autophagy Inhibition Enhances the Antitumor Efficacy of Savolitinib In Vivo

To evaluate whether autophagy inhibition could enhance the antitumor efficacy of savolitinib *in vivo*, a BxPC-3 xenograft model was established in female NCG mice. When tumor volume reached approximately 200 mm^3^, tumor-bearing mice were randomly assigned to five groups (n = 6 per group): vehicle control, savolitinib, CQ, savolitinib plus CQ, and gemcitabine as a positive control. Mice were treated for 21 days, and tumor volume and body weight were monitored twice weekly. Savolitinib was administered at 50 mg/kg by oral gavage once daily [20,21], CQ was administered at 40 mg/kg by intraperitoneal injection once daily [22], and gemcitabine was administered intravenously at 50 mg/kg every two days [23,24], as described in the Section 2. The dosing regimens for savolitinib and CQ were selected based on reported preclinical dosing ranges and tolerability.

**Figure 7:**
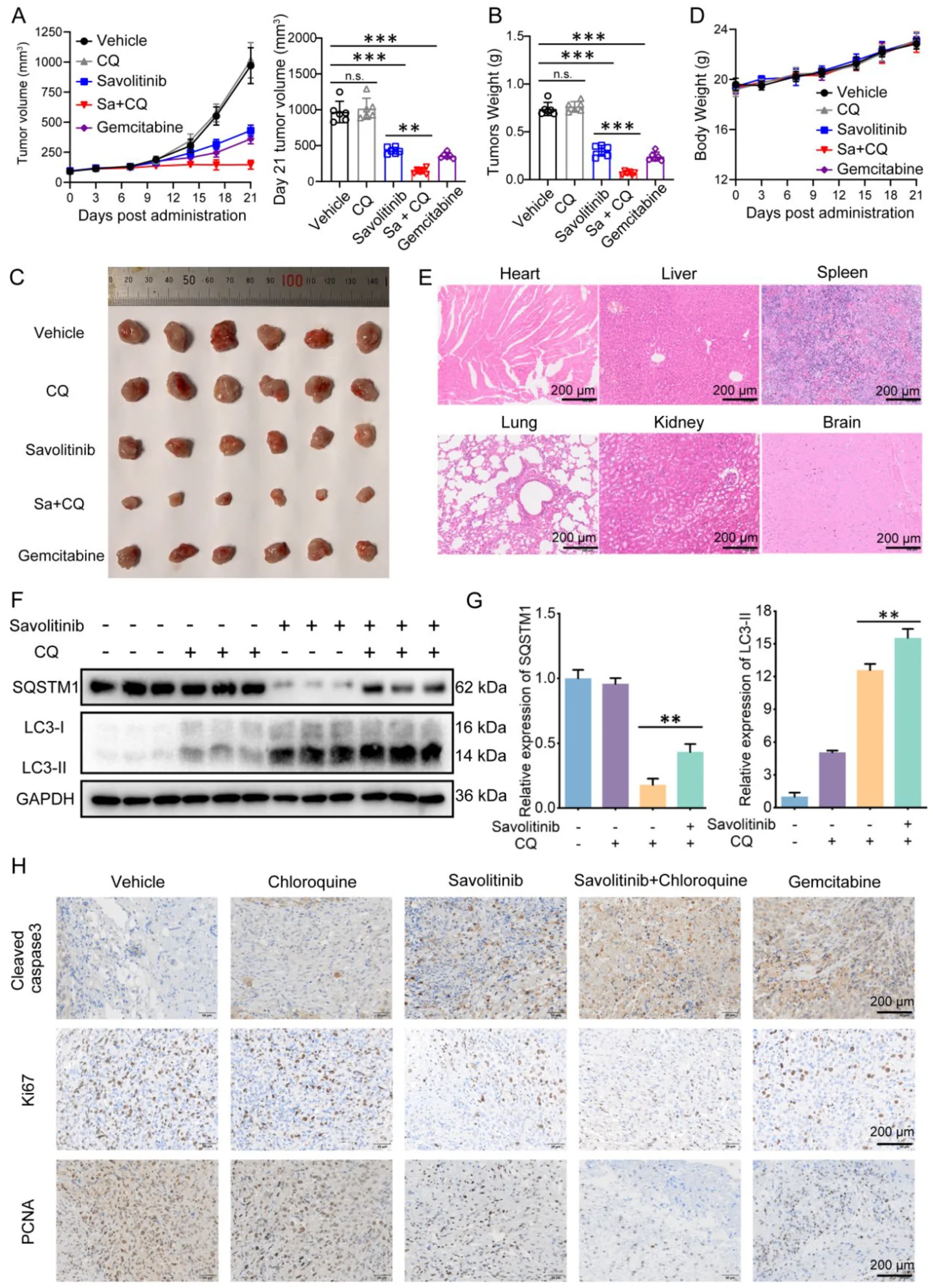
**Combination treatment with savolitinib and chloroquine enhances antitumor efficacy *in vivo*.** (**A**) Tumor volume changes in BxPC-3 xenograft-bearing mice during the 21-day treatment period (n = 6). The inset shows endpoint tumor volumes on day 21 for statistical comparison. (**B**) Excised tumor weights measured at the study endpoint (n = 6). (**C**) Representative images of excised tumors collected from each treatment group at the endpoint. A ruler is shown for scale. (**D**) Body weight changes of tumor-bearing mice during the treatment period (n = 6). (**E**) Representative hematoxylin and eosin (H&E)-stained sections of the heart, liver, spleen, lung, kidney, and brain collected at the end of the 21-day treatment period from mice receiving the combination of savolitinib (50 mg/kg by oral gavage once daily) and chloroquine (40 mg/kg by intraperitoneal injection once daily). Scale bars, 200 μm. (**F**) Representative western blots of autophagy-related proteins in excised tumor tissues. (**G**) Quantification of SQSTM1/p62 and LC3-II levels in tumor tissues shown in (F) (n = 3). (**H**) Representative immunohistochemistry images of cleaved caspase-3, Ki-67, and PCNA in excised tumor tissues. Scale bars, 200 μm. Data are presented as the mean ± SD. Endpoint tumor volume, tumor weight, and protein expression data were analyzed using one-way ANOVA followed by Tukey’s *post hoc* test. Longitudinal tumor-volume and body-weight data were analyzed using two-way repeated-measures ANOVA. n.s., not significant; **, *p* < 0.01; ***, *p* < 0.001.

CQ monotherapy showed minimal antitumor activity and did not significantly differ from the vehicle control group. At the endpoint, the mean tumor volume and tumor weight were 970.7 ± 149.2 mm^3^ and 0.739 ± 0.069 g in the vehicle group, respectively, and 1015.2 ± 147.1 mm^3^ and 0.765 ± 0.052 g in the CQ group. Savolitinib monotherapy significantly suppressed tumor growth, with an endpoint tumor volume of 431.1 ± 44.8 mm^3^ and a tumor weight of 0.302 ± 0.055 g, corresponding to a tumor growth inhibition (TGI) rate of 55.59%. Gemcitabine, used as a positive control, achieved a TGI of 62.79%, with an endpoint tumor volume of 361.1 ± 41.0 mm^3^ and a tumor weight of 0.241 ± 0.045 g. Compared with savolitinib alone, the combination of savolitinib and CQ further enhanced tumor growth inhibition, resulting in an endpoint tumor volume of 145.9 ± 37.1 mm^3^ and a tumor weight of 0.068 ± 0.018 g, corresponding to a TGI of 84.96% (Fig. 7A–C). Endpoint tumor volume comparisons are shown in the inset of Fig. 7A. No significant body weight loss was observed during treatment (Fig. 7D). To preliminarily evaluate the histological safety of the combination treatment, the heart, liver, spleen, lung, kidney, and brain were collected from mice in the savolitinib plus chloroquine group at the end of the 21-day treatment period and subjected to H&E staining. No obvious histopathological abnormalities were observed in the examined organs under the treatment conditions used (Fig. 7E).

To examine whether CQ inhibited autophagy in tumor tissues, Western blot analysis was performed using lysates from excised xenografts. Compared with savolitinib monotherapy, the combination of savolitinib and CQ increased the levels of SQSTM1/p62 and LC3-II in tumor tissues (Fig. 7F,G). The concomitant accumulation of SQSTM1/p62 and LC3-II is consistent with inhibition of autophagic degradation, supporting the blockade of savolitinib-induced autophagic flux by CQ *in vivo*. Immunohistochemistry further showed stronger cleaved caspase-3 staining and reduced Ki-67 and PCNA staining in tumors from the combination group compared with the single-treatment groups (Fig. 7H), suggesting increased apoptosis and decreased tumor cell proliferation. These findings are consistent with the *in vitro* results showing that autophagy inhibition enhances the antitumor effect of savolitinib.

Collectively, these *in vivo* results indicate that inhibition of autophagy with CQ enhances the antitumor efficacy of savolitinib in MET-high pancreatic cancer xenografts. This enhanced therapeutic effect was associated with blockade of autophagic degradation, increased apoptotic signaling, and reduced tumor cell proliferation, without obvious systemic toxicity under the treatment conditions used in this study.

## 4 Discussion

In the present study, we investigated the antitumor effects of the selective MET inhibitor savolitinib and the adaptive cellular responses associated with MET inhibition in pancreatic cancer. Savolitinib preferentially suppressed MET signaling, reduced cell viability, and induced apoptosis in MET-high pancreatic cancer cells. Transcriptomic and experimental analyses further showed that savolitinib elicited an autophagy-associated response accompanied by reduced AKT/mTOR pathway activity. Functional modulation of autophagy suggested that this response primarily served a cytoprotective role, as chloroquine enhanced savolitinib-induced growth inhibition and apoptosis, whereas rapamycin partially attenuated these effects. Consistently, the combination of savolitinib and chloroquine produced greater tumor growth inhibition than either treatment alone in the xenograft model without obvious additional toxicity. Together, these findings suggest that adaptive autophagy may limit the antitumor efficacy of MET inhibition and that co-targeting MET and autophagy warrants further investigation in MET-high pancreatic cancer.

Aberrant HGF/MET signaling has been associated with several malignant characteristics of pancreatic cancer, including enhanced proliferation, invasion, metastatic dissemination, and chemotherapy resistance [7,25]. These observations provide a biological rationale for evaluating MET-directed therapy in molecularly selected pancreatic cancer models. Savolitinib is a highly selective MET inhibitor that has shown antitumor activity in malignancies driven by MET alterations, including non-small cell lung cancer and gastric cancer [11,12]. In the present study, AsPC-1 and BxPC-3 cells, which displayed relatively high MET expression and phosphorylation, were more responsive to savolitinib than MET-low MIA PaCa-2 cells. This differential response was reflected by stronger inhibition of cell viability and more pronounced induction of apoptosis in the MET-high models. However, because only a small panel of pancreatic cancer cell lines was examined, these findings do not establish MET expression alone as a definitive predictive biomarker. Rather, they suggest that the abundance and activation status of MET may influence sensitivity to savolitinib and should be evaluated further in broader preclinical and clinical settings.

A major observation of this study was the coordinated induction of autophagy-associated changes following MET inhibition. RNA-sequencing analysis revealed enrichment of transcriptional programs related to autophagy, lysosomal function, unfolded protein response, p53 signaling, and apoptosis. These findings were supported by autophagy-associated ultrastructural changes observed under transmission electron microscopy, together with increased MDC-positive puncta, LC3-II accumulation, and reduced SQSTM1/p62 protein levels. Moreover, chloroquine co-treatment resulted in further LC3-II accumulation and prevented the reduction of SQSTM1/p62, supporting increased autophagosome formation accompanied by lysosome-dependent substrate turnover rather than a simple blockade of late-stage autophagy [19]. Because no single assay is sufficient to establish autophagic flux, the agreement among the morphological, biochemical, and pharmacological findings provides stronger evidence for an active autophagic response to savolitinib.

SQSTM1 mRNA was increased in the transcriptomic analysis, whereas its protein level decreased after savolitinib treatment. This apparent discrepancy may reflect concurrent stress-responsive transcription of SQSTM1 and enhanced lysosomal degradation of the p62 protein during autophagic flux [26,27]. Thus, transcriptional induction does not necessarily result in protein accumulation when protein turnover is simultaneously increased. Savolitinib also reduced the p-AKT/AKT, p-mTOR/mTOR, p-p70S6K/p70S6K, and p-4E-BP1/4E-BP1 ratios without markedly altering the corresponding total protein levels. Because AKT/mTOR signaling is a major negative regulator of autophagy, these changes suggest that suppression of this pathway may contribute to the autophagic response following MET inhibition [28,29]. Nevertheless, direct pathway-rescue experiments were not performed, and the current results therefore support an association rather than proving that AKT/mTOR suppression is the sole mechanism responsible for savolitinib-induced autophagy.

The functional experiments indicated that this autophagic response was predominantly cytoprotective. Inhibition of late-stage autophagy with chloroquine enhanced the savolitinib-induced reduction in cell viability and increased apoptosis, as shown by Annexin V-FITC/PI staining and the accumulation of cleaved PARP and cleaved caspase-3. Conversely, further stimulation of autophagy with rapamycin partially attenuated the growth-inhibitory effect of savolitinib. These opposing pharmacological effects suggest that autophagy helps MET-high pancreatic cancer cells adapt to the stress caused by MET inhibition. This interpretation is consistent with previous studies showing that targeted anticancer agents can induce autophagy as an adaptive survival response that limits therapeutic activity [29]. The current results demonstrate enhancement of savolitinib activity by chloroquine, although formal pharmacological synergy cannot be concluded because combination-index or response-surface analyses were not performed.

The xenograft experiments further supported the *in vivo* relevance of this combination. Savolitinib alone inhibited BxPC-3 tumor growth, whereas the addition of chloroquine produced a greater reduction in endpoint tumor volume and tumor weight. Tumors from the combination group showed increased LC3-II and SQSTM1/p62 levels, stronger cleaved caspase-3 staining, and reduced Ki-67 and PCNA staining, consistent with impaired autophagic degradation, increased apoptosis, and decreased proliferation. The enhancement observed *in vivo* was more pronounced than that detected in short-term cell culture experiments. One possible explanation is that tumor cells *in vivo* experience prolonged treatment and nutrient-restricted or metabolically stressful conditions that may increase their dependence on autophagy [30,31]. Differences in treatment duration, drug exposure, and interactions with the tumor microenvironment may also contribute to the stronger *in vivo* response. No significant body weight loss or obvious histopathological abnormalities were observed in the examined major organs, indicating no overt systemic toxicity under the treatment conditions used. More detailed pharmacokinetic and toxicological studies would nevertheless be required to characterize the safety of this combination more comprehensively.

This study has several limitations. First, the antitumor effects of savolitinib and chloroquine were evaluated using a limited number of pancreatic cancer cell lines and a single subcutaneous xenograft model. Validation in additional MET-high models, including organoids, orthotopic tumors, and patient-derived xenografts, would improve the generalizability of the findings. Second, chloroquine is not a selective inhibitor of canonical autophagy and can also alter lysosomal acidification and other cellular processes [18,19]. Genetic suppression of essential autophagy-related genes, such as ATG5 or ATG7, would help clarify the specific contribution of autophagy to the enhanced response to savolitinib. Third, the relationship between MET expression, MET activation status, and savolitinib sensitivity remains to be established in larger preclinical and clinically annotated cohorts. Despite these limitations, the present findings support further evaluation of combined MET and autophagy inhibition as a potential therapeutic approach for MET-high pancreatic cancer.

## 5 Conclusion

The present study showed that savolitinib suppressed MET signaling, reduced cell viability, and induced apoptosis in MET-high pancreatic cancer cells, while simultaneously triggering an adaptive autophagy-associated response. Pharmacological inhibition of autophagy with chloroquine enhanced the antitumor effects of savolitinib both *in vitro* and in the BxPC-3 xenograft model without obvious additional toxicity under the tested conditions. These findings suggest that adaptive autophagy may limit the therapeutic efficacy of MET inhibition and provide a rationale for further evaluation of combined MET and autophagy inhibition in molecularly selected pancreatic cancer models.

## Data Availability

The data generated in the current study are available from the corresponding author upon reasonable request. The RNA-seq datasets generated during this study have been deposited in the NCBI Sequence Read Archive (SRA) under BioProject accession number PRJNA1367351.

## Supplementary Materials

The supplementary material is available online at https://www.techscience.com/doi/10.32604/or.2026.084396/s1.

## Abbreviations

4E-BP1	eukaryotic translation initiation factor 4E-binding protein 1
AKT	protein kinase B
ANOVA	analysis of variance
CQ	chloroquine
DEGs	differentially expressed genes
GSEA	gene set enrichment analysis
H&E	hematoxylin and eosin
HGF	hepatocyte growth factor
IHC	immunohistochemistry
LC3	microtubule-associated protein 1 light chain 3
MDC	monodansylcadaverine
MET	mesenchymal–epithelial transition factor
mTOR	mechanistic target of rapamycin
p70S6K	70-kDa ribosomal protein S6 kinase
PARP	poly(ADP-ribose) polymerase
PBS	phosphate-buffered saline
PCA	principal component analysis
PCNA	proliferating cell nuclear antigen
PDAC	pancreatic ductal adenocarcinoma
p-MET	phosphorylated MET
PI	propidium iodide
RNA-seq	RNA sequencing
RSEM	RNA-Seq by Expectation Maximization
SRA	Sequence Read Archive
SQSTM1/p62	sequestosome 1/p62
TEM	transmission electron microscopy
TGI	tumor growth inhibition
UPR	unfolded protein response
VST	variance-stabilizing transformation
