# Supplementary material for: Autophagy Inhibition Enhances the Antitumor Efficacy of MET Targeting in MET-High Pancreatic Cancer
Source: Oncol Res. 2026 Sep 14;34(10):21. doi: 10.32604/or.2026.084396 (PMC13591368; doi:10.32604/or.2026.084396)
Supplement: Supplementary file 1 [file OncolRes-34-84396-s001.zip › TSP_OR_84396_Supplementary_Figures.docx]

**Supplementary Materials**

**Figure S1:** Apoptotic rates of MIA PaCa-2 cells after treatment with savolitinib for 48 h, as determined by annexin V-FITC/PI staining followed by flow cytometry (n = 3). Data are presented as the mean ± SD. Statistical significance was determined by one-way ANOVA followed by Tukey’s *post hoc* test. Comparisons were made with the untreated control group. n.s., not significant.

**Figure S2:** Determination of minimally growth-inhibitory concentrations of autophagy modulators. Cell viability of (**A**) AsPC-1 and (**B**) BxPC-3 cells after treatment with the indicated concentrations of chloroquine (CQ) for 48 h, as assessed by the MTT assay. Cell viability of (**C**) AsPC-1 and (**D**) BxPC-3 cells after treatment with the indicated concentrations of rapamycin for 48 h, as assessed by the MTT assay. Data are presented as the mean ± SD (n = 6). Statistical significance was determined by one-way ANOVA followed by Tukey’s *post hoc* test. Comparisons were made with the untreated control group. The concentrations of 7.5 μM CQ and 60 nM rapamycin did not significantly reduce cell viability in either cell line and were therefore selected for subsequent combination experiments. ns, not significant; **p* < 0.05; ***p* < 0.01; ****p* < 0.001; *****p* < 0.0001.

**Figure S3:** Effect of combining savolitinib with the autophagy inducer rapamycin. (**A**,**B**) Cell viability of (**A**) AsPC-1 and (**B**) BxPC-3 cells after treatment with savolitinib, rapamycin, or their combination for 48 h, as determined by the MTT assay (n = 6). Savolitinib was used at 8 μM, and rapamycin was used at 60 nM. Data are presented as the mean ± SD. Statistical significance was determined by one-way ANOVA followed by Tukey’s *post hoc* test. n.s., not significant; **p* < 0.05.
